# External quality assessment of COVID-19 real time reverse transcription PCR laboratories in India

**DOI:** 10.1371/journal.pone.0263736

**Published:** 2022-02-08

**Authors:** Harmanmeet Kaur, Labanya Mukhopadhyay, Nivedita Gupta, Neeraj Aggarwal, Lucky Sangal, Varsha Potdar, Francis Yesuraj Inbanathan, Jitendra Narayan, Swati Gupta, Salaj Rana, Neetu Vijay, Harpreet Singh, Jasmine Kaur, Vinit Kumar, Nirmal Kaundal, Priya Abraham, Vasanthapuram Ravi

**Affiliations:** 1 Virology Unit, Indian Council of Medical Research, DHR, MoHFW, New Delhi, Delhi, India; 2 WHO South-East Asia Regional Office, New Delhi, Delhi, India; 3 Indian Council of Medical Research-National Institute of Virology, Pune, Maharashtra, India; 4 Division of Biomedical Informatics, Indian Council of Medical Research, New Delhi, Delhi, India; 5 WHO Country Office for India, New Delhi, Delhi, India; 6 National Institute of Mental Health and Neurosciences, Bengaluru, Karnataka, India; All India Institute of Medical Sciences, INDIA

## Abstract

Sudden emergence and rapid spread of COVID-19 created an inevitable need for expansion of the COVID-19 laboratory testing network across the world. The strategy to test-track-treat was advocated for quick detection and containment of the disease. Being the second most populous country in the world, India was challenged to make COVID-19 testing available and accessible in all parts of the country. The molecular laboratory testing network was augmented expeditiously, and number of laboratories was increased from one in January 2020 to 2951 till mid-September, 2021. This rapid expansion warranted the need to have inbuilt systems of quality control/ quality assurance. In addition to the ongoing inter-laboratory quality control (ILQC), India implemented an External Quality Assurance Program (EQAP) with assistance from World Health Organization (WHO) and Royal College of Pathologists, Australasia. Out of the 953 open system rRTPCR laboratories in both public and private sector who participated in the first round of EQAP, 891(93.4%) laboratories obtained a passing score of > = 80%. The satisfactory performance of Indian COVID-19 testing laboratories has boosted the confidence of the public and policy makers in the quality of testing. ILQC and EQAP need to continue to ensure adherence of the testing laboratories to the desired quality standards.

## Introduction

Following the release of SARS-CoV-2 genome sequences in public domain in early January 2020, real time RT-PCR (rRTPCR) assays were developed and shared with researchers all around the world [1]. Indian Council of Medical Research-National Institute of Virology (ICMR-NIV), Pune, the apex laboratory for viral diagnosis in India, adopted the WHO recommended protocol for screening and confirmation of SARS-CoV-2 and started testing suspected cases in India [2]. The first positive case of COVID-19 in the country was diagnosed from Thrissur, Kerala on January 30, 2020 [3].

As the global cases were on a steep rise, the need for quickly augmenting COVID-19 testing capacity in India was realized. For this, 118 Viral Research & Diagnostic Laboratories (VRDLs), operational under the Department of Health Research (DHR) and ICMR were strengthened for COVID-19 diagnosis [2]. Subsequently, other government and private laboratories with capacity to perform molecular testing of RNA viruses were enabled for COVID-19 testing. To expeditiously ramp up testing, public and private laboratories were on-boarded with the help of 14 mentor institutes set up by ICMR [4]. The mentors handheld and guided the laboratories, conducted virtual inspections, trained staff on all laboratory procedures, biosafety and biosecurity practices and monitored their performance. Additionally, all private laboratories were mandated to get accredited by the National Accreditation Board of Laboratories (NABL), which is signatory to International Laboratory Accreditation Cooperation (ILAC) and is an independent accreditation body in India. All these initiatives resulted in expedited augmentation of laboratory testing capacity from a single laboratory in January 2020 to 2951 molecular testing laboratories on September 15,2021 [5]. A total of 663/734 districts in India have a molecular testing laboratory in place.

The COVID-19 testing laboratories in India use a combination of different testing platforms like open system real-time reverse transcription polymerase chain reaction (**rRTPCR**) and closed system **rRTPCR** like TrueNat, CBNAAT (Cartridge Based Nucleic Acid Amplification Test) & other USFDA approved molecular testing platforms. SARS-CoV-2 testing by an open system **rRTPCR** is the gold standard test and is the mainstay of COVID-19 diagnosis in India [6].

**Fig 1:**
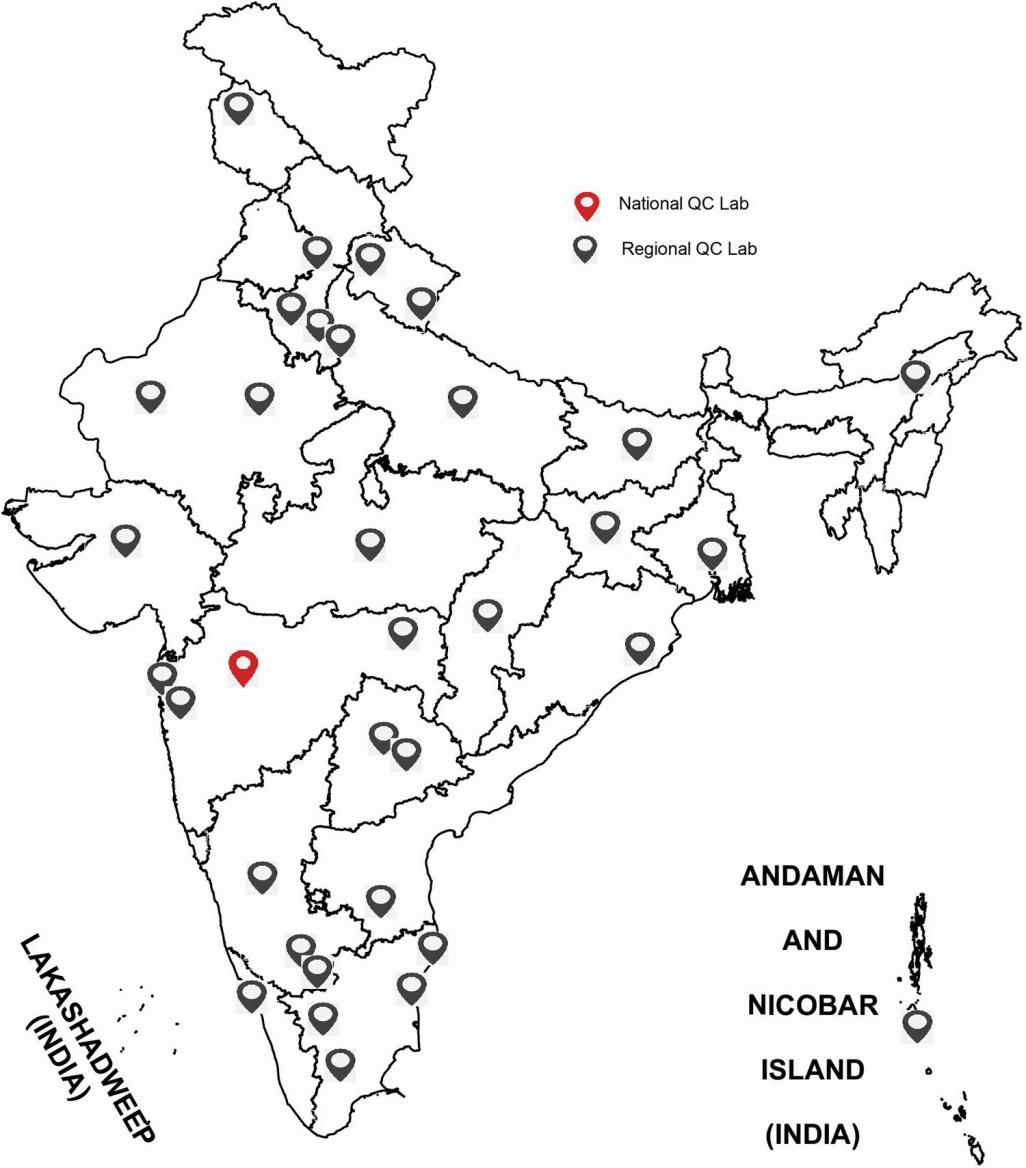
Regional QC laboratories of India.

Laboratories conducting COVID-19 testing by an open system **rRTPCR** use a wide range of test kits with different gene targets and varying quality. Therefore, accuracy, reliability and timeliness of results is always a concern. Sustaining this vast laboratory network with good quality testing was accorded utmost priority since the inception of the pandemic. Since Quality Control (QC) of testing was crucial to build trust and confidence in this vast network of laboratories, this component was inbuilt in the expansion plan of the laboratory network [7]. In order to ensure accuracy and reliability of COVID-19 testing in India, where many laboratories were conducting molecular testing for the first time, ICMR implemented a quarterly inter-laboratory quality control (ILQC) program for **rRTPCR**. A three-tiered structure was created with a National QC laboratory at ICMR-NIV, Pune which is a WHO designated COVID-19 reference laboratory for the South East Asia Region. Thirty-eight geographically well spread laboratories were designated as Regional QC laboratories after attaining 100% QC scores from NIV (Fig 1). All rRTPCR laboratories in public and private sector were linked to the 38 ILQC laboratories with a quarterly sample referral mechanism. During the first quarter of this programme, 93% of the laboratories scored ≥ 90% cut-off QC passing score (manuscript in-press).

To further assess the performance of laboratories conducting COVID-19 testing in India by open system rRTPCR, ICMR was supported by WHO-India and SEARO for conducting External Quality Assurance Program (EQAP) of rRTPCR laboratories through Proficiency Testing (PT) panels prepared by Royal College of Pathologists of Australasia Quality Assurance Programs(RCPAQAP), Australia. Here we describe the performance of 955 COVID-19 rRTPCR laboratories in India in the EQAP.

## Materials and methods

The EQAP was conducted during the period of January to June, 2021.

### Composition of PT panels

A total of 1100 PT panels were centrally received at the WHO reference center at ICMR-NIV, Pune. The panel comprised of 5 samples: 1, 2 and 5 were positive samples, Sample 1 with Ct value (RDRP– 29.9 and N gene –31.4), Sample 2 with Ct value (RDRP- 23.9 and N gene– 25.3) and Sample 5 with Ct value (RDRP—33.4 and N gene– 35.9). Samples 3 and 4 were negative.

#### Panel blinding, standardization, and shipment

The WHO reference laboratory at ICMR-NIV and all the laboratories participating in EQAP were blinded to the results of the PT panel. The panel key was held centrally at ICMR Hq. Five different panels were initially tested at ICMR-NIV, Pune and were found to be 100% concordant qualitatively. Thereafter, the coded panels were shipped to individual laboratories for qualitative testing. All shipments were done under cold chain conditions and as per IATA guidelines. Each shipment box contained a PT panel with 5 samples. Panels were sent to 1095 rRTPCR laboratories of which 941 panels were sent to the laboratories using open system **rRTPCR** and remaining 14 were inadvertently sent to the laboratories using closed system.

### Laboratory assessment and EQA steps

A questionnaire provided by WHO-RCPAQAP was administered to all laboratories before providing them the PT panels (Table 1). The questionnaire helped in understanding the quantum, turnaround time and types of clinical samples of COVID-19 received by them. Further, it also helped to comprehend the scope of testing of different laboratories and the QA/QC mechanisms already in place. EQA exercise comprised of 5 stages–filling in the questionnaire by testing labs, panel dispatch from ICMR-NIV, Pune, panel receipt at the testing lab, result entry by the testing laboratory and report generation by ICMR QA/QC portal. Before receiving the panel, it was mandatory for all the participating laboratories to return the filled-in questionnaire.

**Table 1:** Responses of the testing laboratories to the WHO questionnaire.

S.No	Question	Response of laboratories
1	What are the sources of the specimens for testing in your laboratory?	1011*
2	Is your facility a COVID-only testing laboratory or a polyvalent public health facility?	1011
2.1	COVID-only testing laboratory	394
2.2	Polyvalent public health facility	617
3	What are the common type of specimens accepted for the molecular detection of SARS-CoV-2 in your laboratory?*	1011
3.1	Nasopharyngeal swab	890
3.2	Throat swab	607
3.3	Nasal swab	311
3.4	Tracheal aspirate	48
3.5	BAL	46
3.6	Sputum	31
3.7	Saliva	10
3.8	Blood	9
3.9	Urine	2
3.1	Stool	2
4	Which type(s) of quality control(s) is/are included in the molecular detection of SARS-CoV-2 in you laboratory?*	
4.1	Extraction Control	352
4.2	Internal control (Inhibitor)	669
4.3	Negative control	779
4.4	Positive Control	878
5	Are you testing all specimens coming in?	Yes (1011 laboratories)
6	What is the usual daily sample load/ current maximum daily testing capacity for SAR-CoV-2 in your laboratory? (Daily sample load)	
6.1	<50 samples	197
6.2	51–200 samples	320
6.3	201–500 samples	202
6.4	501–1000 samples	143
6.5	1001–2000 samples	94
6.6	>2000 samples	55
7	What is the usual turnaround time for the molecular detection of SARS-CoV-2 in you laboratory?	
7.1	<24 hours	852
7.2	25–48 hours	157
7.3	49–72 hours	2
8	What is the number of PCR scientists (with a bachelor degree or higher) in your laboratory?	
8.1	One	136
8.2	more than one	875
9	What is the usual daily sample load/ current maximum daily testing capacity for SAR-CoV-2 in your laboratory? (Maximum daily testing capacity)	
9.1	<50 samples	49
9.2	51–200 samples	264
9.3	201–500 samples	251
9.4	501–1000 samples	188
9.5	1001–2000 samples	146
9.6	>2000 samples	113

*Major sources of samples included walk-in patients, home-collection, hospitalized patients, and those attending the out-patient clinic.

#### Reporting and recording results

The ICMR-NIV COVID-19 RT-PCR kit, which detects E, ORF and RDRP genes was used by majority of the laboratories. This multiplex single tube assay uses E gene for screening, ORDF and RDRP for confirmation and beta actin for housekeeping. In-vitro transcribed (IVT) RNA was generated in-house in accordance with established protocols and used as a positive control [7]. Internal validation of the kit was undertaken at NIV, Pune followed by external validation at 10 ICMR recognized laboratories. The kit was found to be satisfactory and was then used by most of the laboratories. Ct value cut-offs for all kits were interpreted as per manufacturers’ instructions. For the ICMR-NIV kit, this cut-off was ≤36.

A centralized QA/QC portal (https://covidqcqa.icmr.org.in/)was developed at ICMR Headquarter, New Delhi for recording PT panel results by testing labs [8]. This portal has a role-based access, enabling confidentiality and complete transparency in the entire QC process as the stakeholders can view results and analysis pertaining to them. Labs were directed to report results of the PT panel on this portal within 7 days of receipt of the samples.

The laboratories were followed up for data entry by a dedicated team of tele-callers.

### Fixing the EQAP passing score

WHO had not defined any passing score for the laboratories participating in the EQAP. In view of this, ICMR constituted an independent expert group to review the EQAP results and to suggest a suitable passing score. The expert group reviewed all raw data along with the results and advised an overall cut-off criterion of 80% concordance as the passing score. It was further specified that all the passing laboratories should essentially score 100% concordance in samples 1–4. The labs not reporting concordant results for sample 5 (weak positive sample in the panel) may be given a score of 80% while those concordant for sample 5 may be given a score of 100%.

## Results

A total of 955(87.2%) out of 1095 laboratories responded within a span of 4–6 weeks. Two laboratories received panels in which 1 or more samples were of insufficient quantity for testing. Therefore, data from 953 laboratories (388 government & 565 private) was analysed and is presented here.

### Response of the laboratories to the WHO questionnaire

Table 1 summarizes the responses of the participating laboratories to the WHO questionnaire (laboratories were permitted to select more than one option). A total of 1011 laboratories responded.

Out of the 1011 laboratories who filled the questionnaire, 61%(617) had existing facilities for rRTPCR test before the pandemic and subsequently adapted to COVID-19 testing after required accreditation, while 39% (394) of the labs were newly established during the pandemic for rRTPCR. Most of the laboratories had a daily testing capacity of up to 200 samples (26.1%), followed by a daily testing capacity of up to 500 samples (24.8%). Majority (98%) of the laboratories were testing nasopharyngeal swab, oropharyngeal swab or nasal swab at the time of conducting EQAP. 1002 (99.1%) of the laboratories had some kind of quality control mechanism in place by using either extraction control, positive control, negative control or an internal (inhibitor) control. All the laboratories were accepting all specimens coming in for COVID-19 testing, provided they had accompanying sample referral forms with pertinent details, and did not meet rejection criteria. Turn-around-time (TAT) for reporting the PT panel results was ≤7 days as stipulated by ICMR for 650 labs (68.2%), whereas 893 labs (93.7%) reported results within 3 weeks of receipt of the PT panel. All the laboratories had at least one trained signatory authority, as per NABL and ICMR guidelines.

### Performance of the laboratories in the EQA exercise

Overall, 891laboratories (93.4%) attained a passing score of ≥80% in the PT panel provided to them.

Table 2 shows sample wise concordance and overall concordance of the laboratories that reported results of the PT panel.

**Table 2:** Sample-wise and overall concordance of COVID-19 testing labs in the EQA exercise.

S.No	N = 953			
A	Sample wise concordance of laboratories	Sample No.	No. of laboratories which correctly detected respective sample	No. of laboratories which could not detect respective sample
		Sample 1	925	28
		Sample 2	944	9
		Sample 3	935	18
		Sample 4	938	15
		Sample 5	667	286
B	100% overall (X)–labs that detected all 5 samples			641 (67.3%) laboratories
C	80% overall and 100% for samples 1–4 (Y)–labs that detected first 4 samples but failed to detect Sample 5			250(26.2%) laboratories
D	Labs that failed to detect one or more samples among Samples 1, 2, 3 and 4			62 (6.5%) laboratories

Total (B+C+D) = 953.

#### Performance of laboratories based on RNA extraction system and amplification platform

496/953 laboratories used the manual method for extraction out of which 447 (90.1%) passed the EQA cut-off score while 49 (9.9%) were below the cut-off.457/953 laboratories used an automated extraction platform. This included 14 laboratories which used closed systems. Out of this, 430 (94.1%), including 13 laboratories with closed systems, passed the EQA cut-off while 27 (5.9%) failed. We concluded that the difference in RNA extraction method did not affect the final EQAP scores.

#### Variation of Ct values of SARS-CoV-2 target genes reported by testing labs

ICMR had initially planned to undertake a qualitative EQA, however since all laboratories were mandated to fill in the Ct values obtained for each sample, ICMR also conducted a quantitative assessment to understand the overall deviation of Ct values among different laboratories.

Most frequently reported confirmatory gene targets by testing laboratories were ORF, RDRP, N and S. The mean Ct values of the laboratories which correctly reported the positive samples (N = 953) are enumerated in Table 3.

**Table 3:** Ct value distribution of SARS-CoV-2 confirmatory genes.

	S gene (6)*			N gene (23)*			ORF gene (733)*			RDRP gene (667)*		
	Mean	SD	95% CI	Mean	SD	95% CI	Mean	SD	95% CI	Mean	SD	95% CI
S1	30.48	2.9	28.2–32.8	30.52	2.53	29.5–31.6	31	2.25	30.8–31.2	31	2.6	30.8–31.2
	S gene (7)*			N gene (25)*			ORF gene (749)*			RDRP gene (698)*		
S2	Mean	SD	95% CI	Mean	SD	95% CI	Mean	SD	95% CI	Mean	SD	95% CI
	26	4.26	22.8–29.2	24.4	2.81	23.3–25.5	25	2.5	24.8–25.2	25	2.6	24.8–25.2
	S gene (4)*			N gene (18)*			ORF gene (513)*			RDRP gene (452)*		
S5	Mean	SD	95% CI	Mean	SD	95% CI	Mean	SD	95% CI	Mean	SD	95% CI
	32	0.55	31.4–32.6	33.7	2.79	31.9–34.5	33	2.23	33.8–34.2	33	2.5	32.8–33.2

*Numbers in brackets indicate the number of labs that have reported results of the confirmatory genes for Samples 1, 2 and 5.

### RT-PCR kits used by testing labs in EQAP

The ICMR-NIV COVID-19 RT-PCR kit was used by 864 labs (90.7%), which detects the E, ORF and RDRP genes. Quality of this in-house kit has been consistent over time and it has been widely used in India since inception of the pandemic, when minimum supply of other diagnostic kits was available. 14 (1.48%) laboratories used closed system rRTPCR, and only 75 (7.8%) labs used other commercial kits, mainly Biomeurix SARS-CoV-2R- Gene, targeting N and RdRP genes, and Trivitron COVIDSURE which detects E, ORDF and RdRP genes. Since the number of laboratories using COVID-19 **rRTPCR** assays other than the ICMR-NIV kit was very small, we did not assess kit wise performance of the testing laboratories, and neither could we analyze any kit specific Ct value deviation for the confirmatory genes.

## Discussion

After emergence of the COVID-19 pandemic, need to augment molecular testing capacity for quick detection and disease containment became inevitable. WHO advised nations to “test-track-treat” to detect every possible case and undertake aggressive contact tracing for limiting disease transmission. Within a short time span of 20 months (January 2020 to September 2021), India scaled up the number of COVID-19 molecular testing laboratories from 1 to 2951 till mid-September 2021. However, fast expansion of the COVID-19 testing laboratories called into question the quality of this network, especially in the private sector. The ILQC initiative taken by ICMR, addressed some quality concerns, but the need for an EQAP to complement the ILQC was realized early on. In ILQC, the participating laboratories have an opportunity to choose the best samples for referral to the designated QC laboratory, thereby reducing the chances of discordance. Availability of positive samples especially in times of low positivity rates is another limitation of ILQC program. EQAP eliminates such hurdles and biases as all laboratories are judged based on uniform parameters and a common set of specimens. With assistance from WHO, India successfully conducted EQAP of its COVID-19 testing laboratories in both Government and private sector.

Out of the 953 laboratories for which EQA results were analysed, 93.4% (891) testing labs were above the cut-off EQA scores of 80% and above. 7.9% of the laboratories were below the cut-off and need close follow-up on improvement of quality. Additionally, the questionnaire filled out by the testing labs highlighted the strengths as well as lacunae in infrastructure and resource limitations which need to be addressed.

Almost equal number of laboratories used manual and automated methods of RNA extraction and no significant difference was found in the performance of the laboratories using different RNA extraction methods. Though EQAP was aimed to assess the laboratories using open-system **rRTPCR** testing for COVID-19, panels were inadvertently sent to 14 labs that use closed method of detection, out of which 13 qualified the exercise.

The EQAP also established that good quality COVID-19 **rRTPCR** kits were being used in the country, and helped in ascertaining at several hundred sites, the performance of the COVID-19 RT-PCR kit that was developed in-house by ICMR-NIV, Pune. None of the laboratories used single target ‘S’ gene assays, thus reducing the chances of false negatives in the backdrop of emerging SARS-CoV-2 variants.

Initially, the EQAP was implemented with the objective of qualitative assessment of the participating laboratories. However, due to recording of Ct values for all samples, the performance was also assessed quantitatively. The narrow confidence intervals for the Ct values of the most commonly detected SARS-CoV-2 confirmatory genes (ORF and RdRP),further highlights the accuracy of COVID-19 testing in the Indian laboratories in both public and private sector (Table 3). The apex virology laboratory of India, ICMR-NIV, Pune also obtained results which closely matched the Ct values provided by WHO.ICMR-NIV has successfully progressed from WHO Influenza NIC to a WHO COVID-19 reference laboratory within a short span of time, and guided COVID-19 testing in the country.

Though conducted methodically, the EQAP in India had a few limitations. 250 (26.2%) laboratories failed to detect the weak positive sample (sample 5). It should, however, be noted that the laboratories that did detect the weak positive specimen in the PT panel, showed high level of accuracy, with narrow confidence intervals for the confirmatory genes. Additionally, only the open system rRTPCR laboratories were included in the EQAP. The open system laboratories cater to a very large proportion of molecular testing in India and hence were prioritized for EQAP in view of limitation of the number of PT panels.

The COVID-19 EQAP will be regularly undertaken in India with inclusion of new as well as underperforming laboratories in subsequent cycles. Influenza QC and EQA has also been initiated in parallel to ensure high quality molecular testing of respiratory pathogens. First cycle of COVID-19 EQAP in India was successfully conducted involving almost a thousand laboratories, even novice ones, with promising results. The EQAP has helped in building confidence in India’s vast COVID-19 diagnostic laboratory network, which is one of the largest in the world. This goes a long way in strengthening quality molecular diagnostic laboratories in the country, which can be utilized and redeployed to effectively deal with future outbreaks.

## Supporting information

S1 Data Individual samples and quesstionnaire responses elaborated in excel file. (XLSX)
